# East and West

**Published:** 1887-12-03

**Authors:** Leslie Keith

**Affiliations:** Author of "The Chilcotes," "Uncle Bob's Niece," etc.


### East and West.

By Leslie Keith.

Author of "The Chilcotes," "Uncle Bob's Niece," etc.

(*Continued from p.* 154.)

X.—On Dangerous Ground,

If one were to hint that Felix Mervyn found his professional walks a trifle more interesting, since they held the possible chance of a meeting with a tall and stately young lady, clad in a gown that severely tried to repress her natural grace, one might be wronging that enthusiastic physician.

It takes a good deal of enthusiasm to live in Baron Street, even if it is your lot to inhabit a house that holds itself aloof in conscious respectability from its neighbours. To be a practitioner in the East End does not imply a distinguished career. Quackery has a good time of it down there, and a new Molière might find occasion for his exquisite satire on the unscrupulous ignorance that prescribes, and the unquestioning faith that swallows "cubic measures" of nastiness, warranted to cure every ache and ail of suffering humanity; so when a young fellow—whose fortune and social position might have permitted him to aspire to Cavendish Square, and the care of aristocratic pulses—chose to throw in his lot with Baron Street, the genteel world very naturally washed its hands of him. A brass-plate in Harley Street and an irreproachable brougham, Mayfair will tolerate; but Mervyn knew himself to be dead and buried when he passed from the west to the east. He accepted his lot for the most part with a very passable philosophy. Whether he liked or disliked to be rediscovered and disinterred by Miss Frankland does not appear, but it was noticeable that he found himself very frequently in her company, and that he manifested a most respectful interest in her questions.

Alicia was still unsatisfied on many points, and, like all youthful reformers, she thought that things might very easily be made different. So they might, if you could get the units to join in concerted action. Units there are in plenty, but they take a long time to make any appreciable change in the mass, especially as each has a theory, which, as a rule, differs from the theory of his neighbour unit.

She found the Bible-woman going her modest way; the Sister, with her tender heart for dirty children; the missionary toiling in paths that are none the less noble because they are obscure from the public gaze; the young priest, curious in vestments and unshaken in his belief in the healing power of the confessional; Felix Mervyn, a better priest than he, with his laugh and his jest; all these excellent people were resolutely fighting the powers of darkness, but Alicia felt that they were not the people who were chiefly wanted.

"They are good already," she said. "They don't want to be enlightened; they are quite alive to what is required here. It is the idle people you must get hold of—the idle and the careless. I should like to make Lady Belgrave spend a week down here, and Lord Mayflower, and Catherine Raddley, and Osman Lloyd, and the others of my select set."

"Not I," cried the doctor, "I have no time for hysterics."

"I think," persisted the young lady, "there must be a remnant of heart in us, if it could be reached; we exhibit it towards our lap-dogs and sometimes towards our children. We are flippant, and hard, and cruel because we have nothing to do, and get so dreadfully tired of each other. We are vulgar; we are more vulgar than Mrs. Jones and Bob Smith and 'Melier and Anner, and the other young ladies of the factory and the slop-shop. Upon reflection, I think it is we who want to be converted and elevated, but you can't do it by preaching sleek sermons to us or sending lecturers to harrow us. We study each other's bonnets during the sermon, and we don't go to the lectures—unless they are very highly spiced and rather naughty—in which case we talk of them at night, and ask for something new next morning. You must bring us down here; you must compel us to see. You must show us 'Arriet and Halice stitching for seventeen hours on a diet of cold tea at work which our maids would scorn to touch; Mrs. Nudge making mantles at an average wage of 2. 9*d*. a week, paying 1*s*. 6*d*. for a lodging, and supporting herself and her child out of the remainder; and Brown getting 4*s*. for the pair of boots for which Tom Spenceley cheerfully gives three guineas. Show us these things, and perhaps—perhaps some of us may believe."

"Heaven forbid!" said the young doctor lightly. "Mayfair, with its lackeys and its maids, its smelling-bottles and fans, its artificialities, airs, and graces, its vapours and spleens, its shrieks and faints, in Bethnal Green!" Yet he looked at her with a new sort of wonder softening the laughter in his eyes.

Here was a young lady who had been the fiercest upholder of her own order, a deserter, a renegade; hot, as every pervert is, and yet hopeless too. He did not like to see the hopelessness in her grey eyes, and yet it was sure to come there sooner or later. The immense amount to be done, and the little that is done, or can be done, paralyses the bravest. What can one little broom—or a hundred, for the matter of that—do to cleanse this Augean stable? Alicia had not gone empty-handed on those visits she and Mary still paid, but she had not given money, which showed a considerable amount of self-denial on her part.

"Collectors and collecting-books have been our ruin," she said. "Five shillings give us a comfortable conscience for a month or two: a sovereign makes us feel virtuous for a whole year. I am going to begin at the other end. I am going to understand first, and then give."

On the whole, it seemed best to her wisdom to spend her money on sending people out of London. Her sympathy and her indignation—for in this proud young lady it often took that form—was chiefly aroused for the country people, the guileless and confiding, who come to London as to an Eldorado where Fortune always smiles; the country workman who tramps up to town, lured by the advertisements of contractors; villagers who find the sphere too narrow at home; workers and idlers whom the mighty heart of the world draws to it by thousands. For the East-ender born and bred she had less pity; the "'appy dosser" begins life in the gutter, and knows and perhaps cares for nothing better; put him in Belgravia to-morrow, and he would speedily convert your "elegant family mansion" into a pig-stye; but the country poor—the village folks, who have had at least clear air, and an untainted heaven above them, what do they not lose physically and morally by the exchange?

"Yet Hodge is our only hope," said Mervyn, looking at the question from a professional point of view; "he keeps up the stamina of the Londoner; he has some physique left, and as a rule can stand up better against disease."

"But you would not keep him here on that account, to deteriorate, to grow reckless and hopeless; to become as sad a problem as his town brothers and sisters?"

"No; I would send him back whence he came, and leave a little more room. But suppose there were no work for him there?"

"There must be," said Alicia.

"It doesn't follow. The chances of employment are certainly greater here. It is a mistake to suppose that the country can claim all the advantages and all the virtues. There are cases of overcrowding in many villages that you couldn't rival in our worst tenements here; and as for rural morality, I'm afraid it will not always bear a strict investigation."

"And yet we boast of our progress," said Alicia, letting her hands fall at her side; "we glory in the splendour of our times, and eighteen hundred years of light and civilisation have brought us no further than this."

In all their talks—and they were many—Mervyn opposed her depression with a gay optimism that sustained him in his self-imposed task.

"Wer'na my hert licht, I wad dee." Don Dismalo is the wrong man to send to the East. The School Board and public opinion were the anchors to which he clung.

"The Education Act is sowing seed for the future; our children and grandchildren will see the fruit, and as for the big, stupid public, it will wake up some morning and discover that we of the East are worth considering. We have been shut in, with the effect of living in a cul-de-sac—always a bad thing morally as well as physically. Wait till the light is let in upon us and you will see."

"When my Mayfair procession begins."

"No," cried Mervyn with vehemence. "I draw the line at Lady Belgrave, and the Honourable Tom, and Osman Lloyd, Esq. I will have none of their High Mightinesses."

"And yet you do not forbid me."

"You," said the young man, suddenly suffering a lack of words. "You are different."

"A month ago I was one of them." Then she went on with a drooping head: "Very likely I am one of them still. If I were to go back to-morrow I should just be the same. I could learn to forget."

"No," he said with conviction. "You would not forget us, any more than we here should forget what you have done for some of us."

But in spite of this consoling assurance Alicia remained sad. Her lately awakened heart was taking a mighty revenge for years of conscious hardness. The sleeping depths were stirred at last. Being a woman, she could not take large or far-reaching views, or console herself with the promise of a brightening future. The present remained, and it was all she had room for in her thoughts.

She had made one or two friends now among her neighbours, and in their homes she was able to witness that curious patience under misfortune, that unembittered acceptance of the inevitable that is characteristic of the poor, and is surely heroic in its way.

"We all get our share of kicks," Mervyn said one day, "but upon my word, when I see the want of distributive justice in the kickings down here, and the manly way in which these poor devils face fortune's buffets, I feel inclined to take off my hat to them."

Among her friends there was one old and palsied doll-maker who specially interested her. She found something almost grotesque in the contrast between the worker and his work—the feeble, trembling artist seated in his pantheon of staring heads and busts. The dolls were the dolls of an archaic age—not the charming creations of Paris to be found nowadays in every nursery—creatures of sawdust, with a vermilion blush, and a glassy stare and a perpetual simper.

"My wife, she makes the bodies, legs, and arms," said this cheerful toiler, "and then there's the cap as has got to be made, too." The cap, as Alicia discovered, was a cunning device to conceal the primitive structure of the head. "I makes the blocks, as you see, and the wax moulds, but my son, 'e puts in the eyes and does the finishing. I'm not equal to it since I took this shaking in my limbs. We begin most mornings by daylight, and it takes us sitting pretty close at it till bedtime to make a livin'."

"And do the shops give you constant orders?" asked Alicia, looking at the dolls with a new respect, inspired by this knowledge of their anatomy.

"Bless you!" said the patriarch, with a smile for her ignorance, "we don't get no orders. We takes 'em round and sells 'em when we can in the shops, but the toys is mostly imported from foreign parts now. Germany, they do say, but if they can make 'em cheaper there, I'm blessed if I know how it's done. There's often days when we're glad to sell them on the streets for what they'll fetch. When we've paid for the stuff there's eightpence of profit on the dozen. And there's a many here that can't make that in the twenty-four hours," he ended, with that readiness to see the excellence of his lot that she found so incredible.

She could and did order dolls to be made for her, dolls by the gross, and what-nots from another optimist over the way, who made the manufacture of this unæsthetic bit of furniture his care, and whose history of a week's tramp in vain search of a purchaser—given with a note of humour too—she felt inclined to print in capitals for distribution among the grumblers of her acquaintance.

"My dinner was a drink of water," said this philosopher, whose small, agile person, and lively, clear-cut features gave him an air of France, "and when I got home at night with the blessed thing on my shoulder, I was that dead beat that I couldn't have eaten the 'vittal' if it had been there to eat—which it weren't. The City Road's a goodish bit of a tramp when you throw in all the turnings off it, right and left, and mind you, that was only one morning's work. But I sold the article in the end, and made three shillings on it, too, by good luck, and that's sixpence more than I've got this year past."

But after all, it was impossible to keep the cabinet-maker or his brother of the dolls, or the match-box makers, or the many cobblers who here do congregate—in constant employment, and an order from a rich young lady meant at best but a week's bread. Alicia looked on at the fight with a heart that ached. The people agonised in the struggle, and counted death kind when it came with its release. David Jardine was wasting day by day, and the sword had entered Mary's soul. The young mechanic from the provinces, with his wife and children, had drifted, as was inevitable, further down the stream; a man must needs be something of a moral hero to resist its force. The youth upstairs, happier in his lot, had died.

Alicia was suffered now to go where she would. Speculation had ceased with regard to her, and expectation of doles had almost ebbed. She did not succeed in many of her attempted friendships, but she was civilly received on the whole; here and there a sullen face scowled at her, and here and there she met with unreserved comment that she privately wished Lady Belgrave could share. She was not one of them, and they knew it in spite of—perhaps in consequence of—the severity of her toilet; the cardboard-box girls and the artificial flower makers wore finery, when they could get it, of an excruciating brilliance. Yet possibly she was saved from open hostility by a rumour that ran through the courts and streets that she was an ambassador with a mission. Anyone who would plead their cause and secure for them benefits to come was welcome to all but the most degraded, who have grown to love the mire in which they wallow.

"And I have been blind all these years," she said one day, unconsciously speaking aloud.

David Jardine was the only other person in the room. He looked up when she spoke, as if her voice had roused him from a dream. His cheeks were hectic and his eyes brilliant; the hands with which he touched his papers trembled.

"I have an offer," he said; "a proposal from a publisher to buy my book." His voice was husky with emotion. "Mary doubted me; she never quite believed in me; but you had faith. You prophesied success, so I tell you first. You are not surprised? You said I should conquer, and you were right."

"No," said Alicia, very gravely, "I am not surprised."

"Ah," said David, with exultation, "I knew it was only a question of time; but it has seemed long. However, all that is forgotten now."

"And this offer—it pleases you, David? It makes you happy?"

"Happy!" he said, and began to walk fiercely up and down. "It is what I have lived for."

Alicia looked at him with a grave wonder. A few weeks earlier her chief feeling would have been scorn; she was not scornful now, but still she wondered. It was a strange ambition to nourish down here, this passionate desire to be heard where everybody else was fighting for a morsel of bread. If he had been the poet of the East-end, the people's voice proclaiming their needs, urging their wrongs on a careless world, she could have understood his thirst for recognition, but all his theme was of peaceful things—of stars and flowers; of hills and valleys; even in love, as he knew it, there was no note of tragedy.

"It is what I have lived for," he cried. "I used to see this day when I lived in Scotland—when I was shut behind that cruel barrier of hills where I could not make my voice heard. Mary will believe now—poor Mary! How could she know? They none of them believed down there; but I knew. I felt it in me." He talked on with growing excitement. Alicia followed him with her grave eyes as he strode up and down the narrow room, his head tossed back, his eyes strangely bright, his lips quivering with eagerness. There was a curious look on the girl's face—was it pity?

"It has made him happy," she said to herself, "and to make anyone happy, even for a little while down here, is worth something. And his success will comfort Mary—that is the best of all."

*(To be continued.)*

